# Therapeutic effects of amniotic fluid-derived mesenchymal stromal cells on lung injury in rats with emphysema

**DOI:** 10.1186/s12931-014-0120-3

**Published:** 2014-10-16

**Authors:** Yaqing Li, Chao Gu, Wulin Xu, Jianping Yan, Yingjie Xia, Yingyu Ma, Chun Chen, Xujun He, Houquan Tao

**Affiliations:** Department of Respiratory Medicine, Zhejiang Provincial People’s Hospital, No. 158, Shangtang Road, Hangzhou, Zhejiang 310014 P.R. China; The Second Clinical Medical College, Zhejiang Chinese Medical University, Hangzhou, Zhejiang 310053 P.R. China; Key Laboratory of Gastroenterology of Zhejiang Province, Zhejiang Provincial People’s Hospital, Hangzhou, Zhejiang 310014 P.R. China

**Keywords:** Mesenchymal stromal cells, Amniotic fluid, Pneumocytes, Chronic obstructive pulmonary disease, Pulmonary emphysema

## Abstract

**Background:**

In chronic obstructive pulmonary disease (COPD), two major pathological changes that occur are the loss of alveolar structure and airspace enlargement. To treat COPD, it is crucial to repair damaged lung tissue and regenerate the lost alveoli. Type II alveolar epithelial cells (AECII) play a vital role in maintaining lung tissue repair, and amniotic fluid-derived mesenchymal stromal cells (AFMSCs) possess the characteristics of regular mesenchymal stromal cells. However, it remains untested whether transplantation of rat AFMSCs (rAFMSCs) might alleviate lung injury caused by emphysema by increasing the expression of surfactant protein (SP)A and SPC and inhibiting AECII apoptosis.

**Methods:**

We analyzed the phenotypic characteristics, differentiation potential, and karyotype of rAFMSCs, which were isolated from pregnant Sprague–Dawley rats. Moreover, we examined the lung morphology and the expression levels of SPA and SPC in rats with emphysema after cigarette-smoke exposure and intratracheal lipopolysaccharide instillation and rAFMSC transplantation. The ability of rAFMSCs to differentiate was measured, and the apoptosis of AECII was evaluated.

**Results:**

In rAFMSCs, the surface antigens CD29, CD44, CD73, CD90, CD105, and CD166 were expressed, but CD14, CD19, CD34, and CD45 were not detected; rAFMSCs also strongly expressed the mRNA of octamer-binding transcription factor 4, and the cells could be induced to differentiate into adipocytes and osteocytes. Furthermore, rAFMSC treatment up-regulated the levels of SPA, SPC, and thyroid transcription factor 1 and inhibited AECII apoptosis, and rAFMSCs appeared to be capable of differentiating into AECII-like cells. Lung injury caused by emphysema was alleviated after rAFMSC treatment.

**Conclusions:**

rAFMSCs might differentiate into AECII-like cells or induce local regeneration of the lung alveolar epithelium *in vivo* after transplantation and thus could be used in COPD treatment and lung regenerative therapy.

## Introduction

Chronic obstructive pulmonary disease (COPD), which is considered to become the third-leading cause of death worldwide by 2020, is currently recognized as a major global public health challenge. In COPD, the major mechanisms underlying chronic airflow limitation are the perpetual loss of alveolar structure, which causes airspace enlargement, and the destruction of the lung parenchyma and the loss of elastic recoil [1]. The loss of alveolar structure and airspace enlargement are key pathological changes in COPD [2], as a result of the development and progression of pulmonary airflow limitation. In the progression of COPD, a critical role is played by the apoptosis of lung epithelial cells, which results in the destruction of the alveolar structure and in emphysema [3]. However, no effective treatment is available for preventing the decline of pulmonary function in patients with COPD. Therefore, the key to treating COPD is to repair damaged lung tissue and regenerate the lost alveoli.

In mammals, the alveolar epithelium is composed of type I alveolar epithelial cells (AECI) and type II alveolar epithelial cells (AECII). AECII are multifunctional secretory cells that are characterized by the presence of morphological lamellar bodies containing pulmonary surfactant proteins (SPs) and lipids. AECII can differentiate into AECI in the alveoli and proliferate to maintain their own number through mitosis [4]. As progenitors of AECI, AECII play vital roles in synthesizing and secreting lung surfactants, which include SPA, SPB, SPC, and SPD, reducing the surface tension of the alveoli, maintaining normal alveolar homeostasis and gas exchange, and improving lung tissue repair [4,5].

Stem cell therapy has attracted considerable attention because of its potential for application in the treatment of COPD, pulmonary fibrosis, cystic fibrosis, and other respiratory diseases [6-8]. By serving as the source of new epithelial cell populations, the resident lung progenitor cells can repair the injured lung epithelium *in vivo* [9]. However, the regenerative capacity of the lung is widely recognized to decline with aging and as a result of extensive damage such as that in COPD; this extensive lung damage might not be repaired appropriately by the endogenous stem niches [10]. Moreover, no evidence is available to suggest that endogenous stem cells can function in alleviating chronic lung disease. However, over the past decade, major breakthroughs in the research on exogenous stem cells have brought new hope for the treatment of COPD. Currently, the exogenous stem cells used mainly include embryonic stem cells (ESCs), bone marrow-derived mesenchymal stromal cells (BMMSCs), and amniotic fluid-derived stromal cells (AFSCs). ESCs are pluripotent stem cells that can be induced to differentiate into various types of cells and ESCs exhibit substantial capacity to proliferate indefinitely [11,12]. For example, ESCs can be induced to differentiate into AECII both *in vitro* and *in vivo* [13,14]. Similarly, BMMSCs alleviate the destruction of lung tissues by also differentiating into AECII *in vivo* [15,16]. However, the challenges involved in acquiring large numbers of BMMSCs from the bone marrow and the low efficiency of their differentiation have restricted research on the use of BMMSCs in regenerative medicine.

Another potential source of cells for lung regeneration *in vivo* are mesenchymal stromal cells (MSCs), which include BMMSCs, amniotic fluid-derived MSCs (AFMSCs), adipose-derived MSCs, and cord blood-derived MSCs; this is because MSCs exhibit the capacity to differentiate into alveolar epithelial cells [17-19]. MSCs have previously been shown to exert beneficial effects on various animal models of respiratory diseases because the cells possess immunomodulatory and anti-inflammatory abilities; the effects of MSCs have been demonstrated in diseases such as COPD [20,21] and asthma [22,23] and in lung fibrosis caused by interstitial lung disease [24] and lung injury caused by acute respiratory distress syndrome [25]. Huh *et al.* [21] reported that MSC-based cell therapy repaired cigarette smoke-induced emphysema in rats after the injection of cells for 2 months. Recently, a placebo-controlled, randomized trial of MSC treatment in patients with moderate-to-severe COPD was published; after the infusion of allogeneic MSCs in COPD patients, no deaths, toxicity, or serious adverse reactions related to the MSC therapy occurred, but the circulating levels of C-reactive protein in the patients were markedly decreased [26].

De Coppi *et al.* [27] reported for the first time that AFSCs can be obtained from discarded amniocentesis specimens and that these cells possess the potential to differentiate widely into neural cells, adipocytes, osteocytes, endotheliocytes, hepatocytes, and cardiomyocytes [28-30]. Thus, AFSCs are recognized as new multipotent stem cells that can be used in regenerative medicine without raising concerns regarding ethical problems or tumorigenesis [31,32]. Furthermore, Carraro *et al.* [33] showed that AFSCs integrated into the embryonic lung tissues of mice, differentiated into lung epithelial cells, and expressed thyroid transcription factor 1 (TTF1) after lung injury *in vivo*. AFMSCs exhibit the characteristics of MSCs *in vitro*, by expressing molecules such as CD73, CD90, CD105, and CD166 [34,35]. AFMSCs were previously shown to be capable of differentiating into AECII-like cells *in vitro*, which indicated that AFMSCs can potentially be used in lung-tissue regenerative therapy [35]. Moreover, rat AFMSCs (rAFMSCs) were shown to secrete neurotrophic factors and thereby promote the regeneration of the injured sciatic nerve [36] and to over-express the interleukin-1 receptor antagonist and thus improve hepatic function *in vivo* in rats with fulminant hepatic failure [37]. However, whether rAFMSCs can exert therapeutic effects on lung injury caused by emphysema is unknown. In this study, we transplanted rAFMSCs into rats with emphysema and then investigated whether the rAFMSCs integrated into lung tissue, expressed AECII-specific markers, inhibited AECII apoptosis, and alleviated lung injury caused by emphysema.

## Materials and methods

### Animals

We purchased 15 pregnant Sprague–Dawley rats (body weight, 300 ~ 350 g, at 12–14 days of pregnancy) and 60 female Sprague–Dawley rats (body weight, 180–200 g) from Shanghai SLAC Laboratory Animal Co., Ltd (Shanghai, China). All animal protocols were approved by the Ethics Committee of Zhejiang Provincial People’s Hospital.

### Isolation and culture of rAFMSCs

We isolated rAFMSCs from pregnant Sprague–Dawley rats as described by Pan *et al.* [36]. The rats were deeply anesthetized using 10.0% chloral hydrate, and then 15 independent amniotic fluid samples (2 mL each) were harvested from fetal male rats by using 22-gauge needles. Each sample was filtered through a 200-mesh filter and centrifuged for 10 min at 1,500 rpm. The cells were resuspended in low-glucose Dulbecco’s modified Eagle’s medium (DMEM; Gibco, Carlsbad, CA, USA) supplemented with 20% fetal bovine serum (FBS; HyClone, Logan, UT, USA) and 4 ng/mL basic fibroblast growth factor (bFGF; Sigma, St. Louis, MO, USA), and then maintained in 6-well plates and grown in a humidified incubator at 37°C with 5% CO_2_. To remove non-adherent cells, the supernatants were replaced for the first time after 5 days of culture. The adherent cell clones that exhibited spindle-shaped growth were detached using cell scrapers and transferred to new 6-well plates. When the cells reached 70%-80% confluence, they were detached using 0.25% trypsin (Sigma) and cultivated in 25-cm^2^ culture flasks (Corning Inc.-Life Science, Oneonta, NY, USA) and labeled as P1 (passage 1). We used P3 cells to conduct the experiments described next. To measure the expression of octamer-binding transcription factor 4 (*Oct-4*) mRNA, we used reverse-transcription polymerase chain reaction (RT-PCR). Rat lung fibroblasts (RFL-6; ATCC, Manassas, VA, USA) were used as the negative control.

### Flow cytometry analysis

We used flow cytometry in order to detect the specific surface antigens of rAFMSCs (P3). Cells in suspension were incubated with fluorescein isothiocyanate (FITC)-conjugated antibodies against CD14, CD19, CD34, CD44, CD45, and CD90, and phycoerythrin (PE)-conjugated antibodies against CD29, CD73, CD105, and CD166 (BD Biosciences, San Diego, CA, USA) at 4°C for 1.5 h. Thereafter, the cells were analyzed using a flow cytometer (Guava EasyCyte; Millipore, Billerica, MA, USA); the related isotype controls were used as the negative control.

### Differentiation potential of rAFMSCs

To induce rAFMSCs to differentiate into adipogenic and osteogenic cell lineages, cells were cultured for 3 weeks in either an adipogenic medium [α-modified minimum essential medium (α-MEM; Gibco) containing 10% FBS and 1 μmol/L dexamethasone, 5 μg/mL insulin, 0.5 mmol/L isobutylmethylxanthine, and 60 μmol/L indomethacin (all 4 reagents from Sigma)], or an osteogenic medium (α-MEM containing 10% FBS and 0.1 μmol/L dexamethasone, 10 mmol/L β-glycerol phosphate, and 50 μmol/L ascorbate; Sigma). In the case of adipogenic differentiation, intracellular accumulation of lipid droplets was examined by means of Oil Red O staining, whereas in the case of osteogenic differentiation, alizarin red staining was used to observe calcium mineralization.

### Chromosomal analysis

We prepared P3 and P15 rAFMSCs for the purpose of chromosomal analysis, as previously described [38]. The rAFMSCs were treated with 0.1 mg/mL colchicine (Sigma) at 37°C for 3 h and then incubated with 0.06 mol/L KCl at 37°C for 30 min. Next, metaphase chromosomes were analyzed using G banding and Giemsa staining; we examined a minimum of 20 metaphases in each sample by using an 80i microscope (Nikon, Tokyo, Japan) and then analyzed them by using Genikon software (Nikon).

### Animal model and rAFMSC transplantation

We maintained 60 female Sprague–Dawley rats at 21°C-25°C and 40%-60% relative humidity in a 12-h light/dark cycle and provided them with water and food *ad libitum*. After 1 week of conditioning, the rats were randomly sorted into 4 groups (15 rats/group), control (group I), emphysema (group II), emphysema + rAFMSC transplantation for 20 days (group III), and emphysema + rAFMSC transplantation for 40 days (group IV).

A custom-designed cigarette-smoke chamber [39] and lipopolysaccharide (LPS; Sigma) stimuli were used for generating the rat model of emphysema [40]. In this study, we used commercially available cigarettes (XiongShi; China Tobacco Zhejiang Industrial. Co., LTD, China) that contained 0.7 mg of nicotine and 8 mg of tar per cigarette. We performed the cigarette-smoke exposure and intratracheal LPS instillation as follows, rats in groups II, III, and IV were placed in the cigarette-smoke chamber (60 cm × 50 cm × 40 cm), and after the rats had settled, the smoke of 5 cigarettes was successively delivered in 12 min. After an interval of 10 min, the smoke of 5 new cigarettes was delivered into the chamber. The rats were exposed to 20 cigarettes over 90 min once a day for each smoke exposure and for 7 days per week for 12 weeks. During the exposure, the concentration of carbon monoxide was maintained almost constant, after 30, 60, and 90 min exposures, the concentrations were 402 ± 19, 399 ± 12, and 408 ± 14 ppm, respectively, as measured using a carbon monoxide detector (CTB-999; INDUSTRIAL SCIENTIFIC. Co., LTD, Shanghai, China). On the last day of the 4^th^ and 8^th^ weeks, each rat in groups II, III, and IV was temporarily anesthetized with 5.0% isoflurane, after which the rats were intratracheally instilled with 200 μL of 1 μg/μL LPS in sterile phosphate-buffered solution (PBS). The rats in group I received clean air throughout the experimental period.

Y-chromosome-positive rAFMSCs were transplanted into the rats of the emphysema groups. Each rat in groups III and IV was intratracheally instilled with rAFMSCs (4 × 10^6^ in 200 μL of PBS), and after transplantation for 20 days and 40 days, the rats were sacrificed by intraperitoneal injection of 10.0% chloral hydrate. The left lungs were removed and fixed in 4% paraformaldehyde for use in histological examinations and immunohistochemical and immunofluorescence staining. The right lungs were stored at −80°C for use in PCR and western blotting analyses.

### Histological examinations

The left lungs were perfused intratracheally with 4% paraformaldehyde at a constant pressure of 25 cm H_2_O for 1 h, and then immersed in paraformaldehyde for 24 h to fix them completely. After fixation, the left-lung blocks were embedded in paraffin and cut into 4-μm-thick sections. Three discontinuous paraffin-embedded sections of each lung-tissue sample were stained with hematoxylin and eosin (H&E) in order to assess the morphological changes in the lungs. We examined 5 fields of view in each of the 3 sections from each lung sample by using a light microscope (Olympus, Tokyo, Japan), and we avoided selecting fields containing bronchi and large blood vessels. We obtained the mean linear intercept (MLI), which indicates the average distance between opposing walls of a single alveolus and is a measure of pulmonary airspace enlargement [41]. Moreover, we obtained the mean alveolar airspace (MAA), which is also a measure of pulmonary airspace enlargement [39].

### Quantitative real-time PCR

Total RNA was extracted from each right-lung sample by using TRIzol reagent (Invitrogen, Carlsbad, CA, USA) according to the manufacturer’s protocols. The final RNA purity and concentrations were determined using a spectrophotometer. Next, cDNA was synthesized from the total RNA by using the PrimeScript^TM^ RT reagent Kit with gDNA Eraser (TaKaRa, Otsu, Japan) according to the manufacturer’s instructions. Quantitative real-time PCR analysis was performed using previously described parameters [35] and real-time PCR amplification equipment (MX3000P; Agilent Technologies, Inc., Santa Clara, CA, USA). The following primers were used (Invitrogen), *SPA* (GenBank, NM_001270647.1), 5'-TCGGTGTCCCAGGATTTAG-3' (forward) and 5'-CAGGGTGGCTGCTGTTAGT-3' (reverse); *SPC* (GenBank, NM_017342.2), 5'-CAGACACCATCGCTACCTT-3' (forward) and 5'-TAGCCAAAGCCTCAAGACT-3' (reverse); *TTF1* (GenBank, XM_006233882.1), 5'-CATCAGATTCTGCAAACAATGG-3' (forward) and 5'-AGGAGTCTGGCCTTCAATCA-3' (reverse); *GAPDH* (GenBank, NM_017008.4), 5'-GTTCAACGGCACAGTCAAG-3' (forward) and 5'-GCCAGTAGACTCCACGACAT-3' (reverse). All analyses were performed in triplicate. Glyceraldehyde-3-phosphate dehydrogenase (*GAPDH*) was used as a reference gene. The relative expression of *SPA*, *SPC*, and *TTF1* mRNAs was calculated using the 2^−ΔΔCt^ method.

### Immunohistochemistry

We performed immunohistochemical staining in order to examine the expression of SPA and SPC proteins in formalin-fixed, paraffin-embedded sections of each left-lung sample. Sections were deparaffinized and rehydrated using a graded series of ethanol, and this was followed by a high-pressure antigen-retrieval step. Endogenous peroxidase activity was blocked by incubating sections with 3% hydrogen peroxide for 15 min. The sections were next blocked with 10% goat serum (ZSGB-BIO, Beijing, China) for 1 h, incubated (overnight, 4°C) with rabbit anti-rat SPA or SPC primary antibodies (1:500; Santa Cruz Biotechnology Inc., Santa Cruz, CA, USA), and then stained with an anti-rabbit IgG secondary antibody (SP-9000 kit; ZSGB-BIO) and 3,3'-diaminobenzidine (DAB; ZSGB-BIO). Hematoxylin was used for counterstaining. To calculate the percentages of SPA- and SPC-positive cells, we used a light microscope and counted the SPA- and SPC-positive cells present among a total of 400 cells in 10 different lung samples, and we used 10 randomly selected alveolus fields in the case of each lung sample.

### Western blotting

Proteins were extracted from each right-lung sample by homogenizing the samples in ice-cold lysis buffer (50 mmol/L Tris, pH 7.4, 150 mmol/L NaCl, 0.1% sodium dodecyl sulfate, 1 mmol/L EDTA, 1% sodium deoxycholate, and 1% Triton X-100; Invitrogen) containing a protease-inhibitor cocktail (1 mmol/L phenylmethylsulfonyl fluoride, 1 mg/L leupeptin, and 1 mg/L aprotinin; Beyotime, Nantong, China). The homogenates were centrifuged for 15 min at 12,000 rpm and the supernatants were collected. The protein concentration in samples was determined by using a micro BCA protein assay kit (Pierce, Rockford, IL, USA) according to the manufacturer’s protocol. Next, equal amounts of protein (20 μg) from each sample were heated at 100°C for 5 min and then separated by electrophoresing them on 8% sodium dodecyl sulfate-polyacrylamide gels. The separated proteins were electrotransferred to nitrocellulose membranes and blocked with Tris-buffered saline containing Tween-20 (TBS-T) and 5% bovine serum albumin (BSA) for 2 h at room temperature. The membranes were then incubated with primary antibodies against SPA and SPC (1:200 in TBS-T; Santa Cruz) and β-actin (1:1000; Abcam, Cambridge, MA, USA) overnight at 4°C on an orbital shaker. After washing 3 times for 10 min each in TBS-T, the membranes were incubated with horseradish peroxidase-conjugated goat anti-rabbit IgG (H + L) (Pierce) for 1 h at room temperature on an orbital shaker. After washing 5 times with TBS-T, immunoreactive bands on the membrane were detected using an enhanced chemiluminescence solution (Amersham Pharmacia Biotech, Buckinghamshire, UK), visualized by means of X-ray-film exposure, and analyzed using an UVP-GDS8000 gel-analysis system (Ultra-Violet Products Ltd., Cambridge, UK). Protein expression levels were analyzed by performing densitometry and the values were normalized relative to those measured for β-actin, which was used as the loading control.

### Reverse-transcription PCR

Total RNA was extracted from each right-lung sample as described above and PCR was performed using previously described parameters [42] and PCR amplification equipment (MJ-PTC200; Bio-Rad, Hercules, CA, USA). The following primers were used, *Oct-4* (GenBank, NM_001009178.2), 5'-GAAGGATGTGGTCCGAGTGT-3' (forward) and 5'-GTGAAGTGAGGGCTCCCATA-3' (reverse); Sex determining region Y (*Sry*) (GenBank, NM_012772.1), 5'-GCAATGGGACAACAACCTAC-3' (forward) and 5'-TGTTTCTGCTGTAGTGGGTATC-3' (reverse); *GAPDH* (GenBank, NM_017008.4), 5'-GTTCAACGGCACAGTCAAG-3' (forward) and 5'-GCCAGTAGACTCCACGACAT-3' (reverse). *GAPDH* was amplified for the purpose of normalizing the target genes in each group. The *Oct-4*, *Sry*, and *GAPDH* PCR products were 183, 138, and 136 bp long, respectively. The amplified PCR products were electrophoretically separated on 1.5% agarose gels, and the band densities were determined using the UVP-GDS8000 gel-analysis system.

### TUNEL/SPC immunofluorescence staining

AECII apoptosis in each group was detected using terminal deoxynucleotidyl transferase dUTP-mediated nick-end labeling (TUNEL; Beyotime)/SPC double-staining as described [43]. Paraffin-embedded left-lung sections were deparaffinized and rehydrated and then subjected to the high-pressure antigen-retrieval step and proteinase K (20 μg/mL) treatment for 20 min. The sections were next incubated with the rabbit anti-rat SPC primary antibody (1:300; Santa Cruz) overnight at 4°C and then with the FITC-conjugated anti-rabbit IgG secondary antibody (1:500; Santa Cruz) for 1 h at 37°C, and this was followed by incubation with 50 μL of the TUNEL reaction mixture at 37°C for 1 h in a humidified chamber. Lastly, the sections were counterstained with 4',6-diamino-2-phenylindole (DAPI; Sigma) and examined using a fluorescence microscope (Axio vert; Carl Zeiss, Jena, Germany). We randomly selected 100 images from 10 different lung samples, and then chose 10 discontinuous areas of each lung sample for the analysis. The percentage of AECII apoptosis was determined by dividing the number of apoptotic AECII, which were positive for both TUNEL and SPC, by the total number of DAPI-positive cells.

### *In situ* hybridization and immunofluorescence

Fluorescence *in situ* hybridization (FISH) for Y chromosome (Y-FISH) was performed as described [33] by using the *Sry* mRNA *in situ* hybridization detection kit (BOSTER, Wuhan, China) according to the manufacturer’s protocols. Paraffin-embedded left-lung sections were deparaffinized and rehydrated, blocked with 3% hydrogen peroxide for 10 min, and digested with proteases (diluted using 3% citric acid) for 15 min at room temperature. The sections were rinsed 3 times in PBS, and then 20 μL of the prehybridization solution was incubated with each section for 4 h at 42°C in a humidified chamber; to perform hybridization, we incubated 20 μL of *Sry* oligonucleotide probes with the sections overnight at 42°C, with the samples being sealed with coverslips (BOSTER). After removing the coverslips, the sections were immersed sequentially in 2× standard saline citrate (SSC; BOSTER) for 10 min, 0.5× SSC for 15 min, 0.2× SSC for 30 min (all at 37°C), and then incubated with cy3-conjugated rat IgG streptavidin-biotin complex (SABC-cy3; Eton Bioscience Inc., San Diego, CA, USA) for 45 min at 37°C. Lastly, the sections were counterstained with DAPI and examined using an IX71 fluorescence microscope (Olympus). To combine this hybridization with immunostaining for SPC, sections were incubated with the anti-rat SPC primary antibody (1:500; Santa Cruz) overnight at 4°C and then with the FITC-conjugated anti-rabbit IgG secondary antibody (1:500; Santa Cruz) for 1 h at room temperature. We randomly selected 100 images from 10 different lung samples and analyzed 10 discontinuous areas in each lung sample. The percentage of differentiation was determined by counting the cells that were positive for both Y-FISH and SPC and dividing this number by the total number of DAPI-positive cells.

### Statistical analysis

Data are expressed as means ± SEM and were analyzed for statistical significance by means of one-way analysis of variance (ANOVA) and independent-sample *t*-tests. Multiple comparisons in ANOVA were performed using the Student-Newman-Keuls test. *P* < 0.05 was considered statistically significant.

## Results

### Culturing rAFMSCs

**Figure 1:**
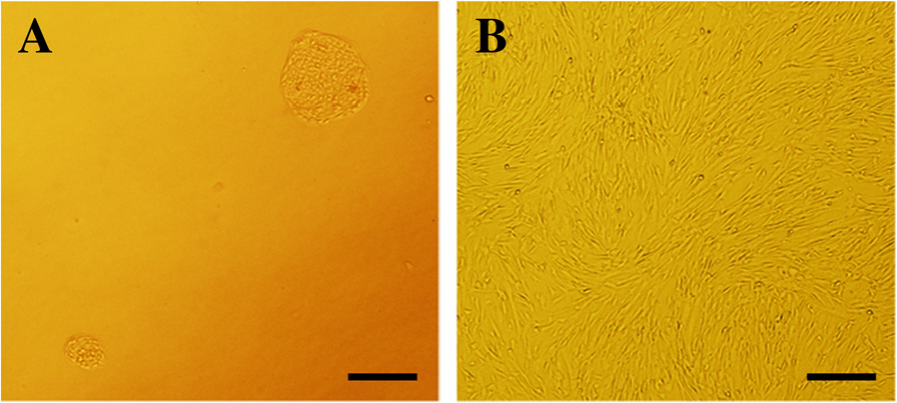
**Culture of rAFMSCs. (A)** Morphological characterization of rAFMSCs. After rAFMSCs were cultured 5 days, colonies began to appear. **(B)** Morphology of rAFMSCs. After 2 weeks of culture, the cells (P3) were spindle shaped and exhibited spiral growth. Scale bars, 200 μm.

Colonies of rAFMSCs began to appear after the cells had been cultured for 5 days in DMEM medium containing 20% FBS and 4 ng/mL bFGF (Figure 1A). When the cells reached 80%-90% confluence, spindle-shaped cells (P3) became dominant and they grew spirally after 2 weeks of culture (Figure 1B).

### Phenotypic characterization of rAFMSCs

**Figure 2:**
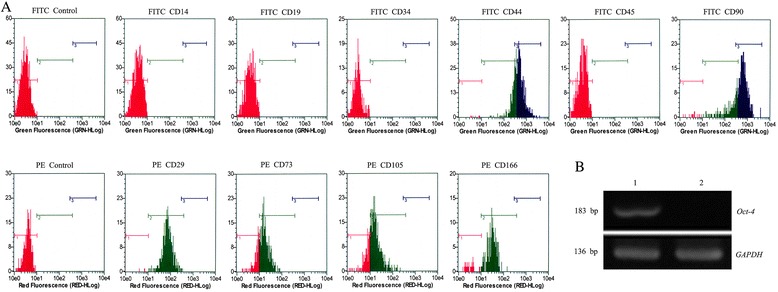
**Phenotypes and** ***Oct-4*** **mRNA expression of rAFMSCs. (A)** The rAFMSCs were phenotypically characterized by means of flow cytometry. The relevant isotype controls were used as the negative control. The surface antigens CD29, CD44, CD73, CD90, CD105, and CD166 were expressed in P3 rAFMSCs, but CD14, CD19, CD34, and CD45 were not. **(B)** *Oct-4* mRNA expression in rAFMSCs was analyzed by performing RT-PCR. Lane 1, rAFMSCs; Lane 2, negative control (rat lung fibroblasts).

The surface antigenic characteristics of rAFMSCs at P3 were analyzed using flow cytometry. The results revealed that the cells were positive for these surface antigens, CD29 (99.4% ± 0.4%), CD44 (99.3% ± 0.4%), CD73 (76.7% ± 4.5%), CD90 (97.5% ± 1.6%), CD105 (74.5% ± 4.7%), and CD166 (89.3% ± 3.1%); by contrast, they were negative (0%) for CD14, CD19, CD34, and CD45 (Figure 2A). Moreover, RT-PCR results showed that the *Oct-4* mRNA was strongly expressed by the rAFMSCs (Figure 2B).

### Differentiation potential of rAFMSCs

**Figure 3:**
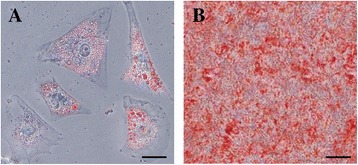
**Differentiation potential of rAFMSCs. (A)** Adipogenic differentiation, cells positive for Oil Red O staining. **(B)** Osteogenic differentiation, cells positive for alizarin red staining. Scale bars, 50 μm **(A)** and 200 μm **(B)**.

To evaluate the differentiation potential of rAFMSCs, cells at P3 were induced to differentiate into adipocytes and osteocytes. After rAFMSCs were cultured in an adipogenic medium for 3 weeks, Oil Red O-positive intracellular lipid droplets were observed (Figure 3A). Similarly, after rAFMSCs were cultured in an osteogenic medium for 3 weeks, most of the differentiated cells appeared cubical and exhibited a dull-red alizarin-red staining that indicated calcium mineralization (Figure 3B).

### Chromosomal analysis

To identify the karyotype and to confirm chromosomal stability, we performed karyotype analysis on P3 and P15 rAFMSCs. The results revealed that rAFMSCs contained a normal diploid number of chromosomes (2n = 42) and maintained a normal karyotype (Y-chromosome-positive) at these distinct passages (data not shown).

### Histopathological changes

**Figure 4:**
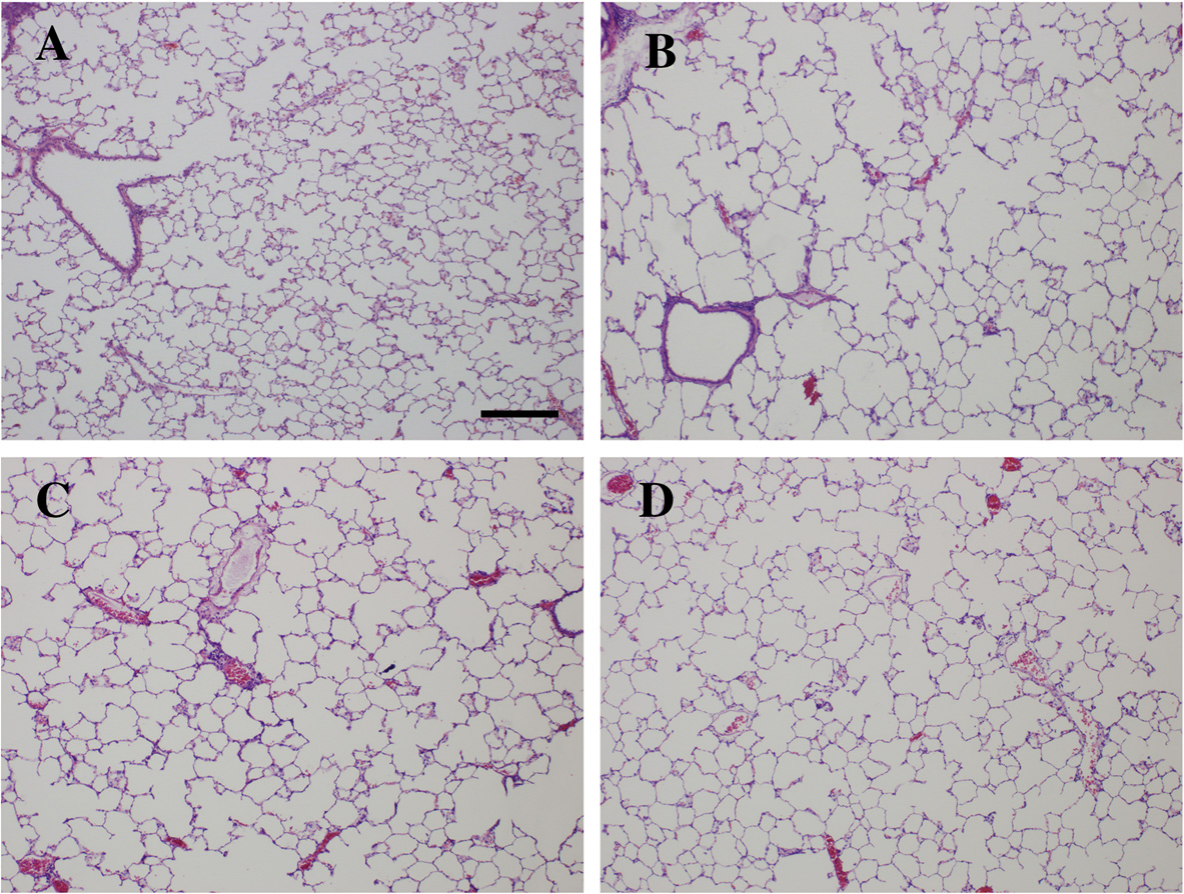
**Histopathologic changes of lung tissues in rats of various groups. (A)** Control group (group I). **(B)** Emphysema group (group II). Airspace was markedly enlarged and the amount of alveoli was decreased after cigarette-smoke exposure and intratracheal LPS instillation. **(C)** Emphysema + rAFMSC transplantation for 20 days (group III). The features of emphysema were partly diminished after rAFMSC transplantation for 20 days. **(D)** Emphysema + rAFMSC transplantation for 40 days (group IV). The features of emphysema were decreased substantially after rAFMSC transplantation for 40 days. Scale bars, 200 μm.

After 12 weeks of cigarette-smoke exposure and 2 intratracheal LPS instillations, the airspace was enlarged markedly and the number of alveoli was decreased in the lung samples of the rats of the emphysema group (group II) (Figure 4B). When compared with the samples from group I (Figure 4A), the samples from group II exhibited numerous merged alveoli and the formation of a few bullae, which was consistent with the pathological characteristics of emphysema. However, both of the emphysema characteristics were partly alleviated after rAFMSC transplantation, as shown in Figure 4C and especially in Figure 4D. Quantitative analyses of lung histomorphology revealed that the MLI and MAA of group II were significantly higher than those of the control group (95% confidence intervals, MLI, 51.42 ~ 64.82; MAA, 8219.06 ~ 9136.90; *P* < 0.01). However, the MLI and MAA of groups III and IV were significantly lower than those of group II (95% confidence intervals, group III, MLI, −35.47 ~ −22.07; MAA, −3900.76 ~ −2982.92; group IV, MLI, −48.42 ~ −35.02; MAA, −5648.48 ~ −4730.65; *P* < 0.01), especially in group IV (95% confidence intervals, MLI, −19.93 ~ −5.94; MAA, −2227.04 ~ −1268.39; *P* < 0.01; Table 1).

**Table 1:** **Quantitative analyses of lung histomorphology**

**Group**	**MLI (μm)**	**MAA (μm** ^**2**^ **)**
I	56.53 ± 3.35	4872.28 ± 194.47
II	114.66 ± 12.22*	13550.27 ± 619.55*
III	85.88 ± 6.07**	10108.42 ± 691.21**
IV	72.94 ± 4.60 **^#^	8360.70 ± 445.85**^#^

Measurement of the parameters MLI and MAA revealed that rAFMSCs could alleviate lung injury in rats with emphysema after being transplanted for 20 and 40 days. Group I, control. Group II, emphysema. Group III, emphysema + rAFMSC transplantation for 20 days. Group IV, emphysema + rAFMSC transplantation for 40 days. Values are presented as means ± SEM (n = 10). *P < 0.01 versus group I, **P < 0.01 versus group II, ^#^ *P* < 0.01 versus group III. MLI = Mean linear intercept; MAA = mean alveolar airspace.

### Expression of *SPA*, *SPC*, and *TTF1* mRNAs

**Figure 5:**
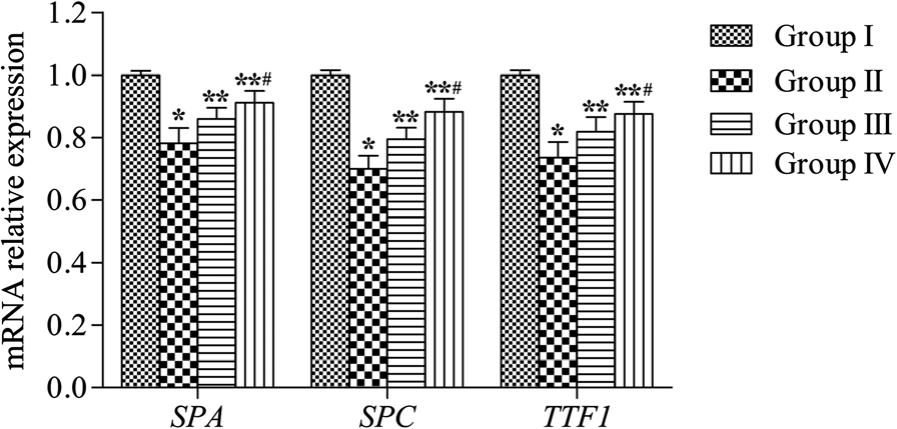
***SPA*** **,** ***SPC*** **, and** ***TTF1*** **mRNA expression in lung tissues.** *SPA*, *SPC*, and *TTF1* mRNA expression levels in lung tissues were determined using quantitative real-time PCR. The relative expression of *SPA*, *SPC*, and *TTF1* mRNAs was calculated using the 2^−ΔΔCt^ method. Values are presented as average expression levels of the mRNAs (means ± SEM, n = 12); *P < 0.05 versus group I, **P < 0.05 versus group II, ^#^ *P* < 0.05 versus group III.

High levels of *SPA*, *SPC*, and *TTF1* mRNAs were expressed in samples from group I (control group), but these levels were markedly lower by comparison in the emphysema group (group II) (95% confidence intervals, SPA, −0.23 ~ −0.17; SPC, −0.30 ~ −0.28; TTF1, −0.32 ~ −0.28; *P* < 0.05). After rAFMSC transplantation (groups III and IV), the expression levels of *SPA*, *SPC*, and *TTF1* mRNAs were significantly higher than those measured in the case of group II (95% confidence intervals, group III, SPA, 0.03 ~ 0.09; SPC, 0.02 ~ 0.07; TTF1, 0.06 ~ 0.12; group IV, SPA, 0.06 ~ 0.12; SPC, 0.11 ~ 0.15; TTF1, 0.12 ~ 0.17; *P* < 0.05). Compared with rAFMSC transplantation for 20 days (group III), the transplantation for 40 days (group IV) significantly increased the expression of *SPA*, *SPC*, and *TTF1* mRNAs (95% confidence intervals, SPA, 0.01 ~ 0.07; SPC, 0.07 ~ 0.14; TTF1, 0.01 ~ 0.06; *P* < 0.05) (Figure 5).

### Immunohistochemical analysis of SPA and SPC expression

**Figure 6:**
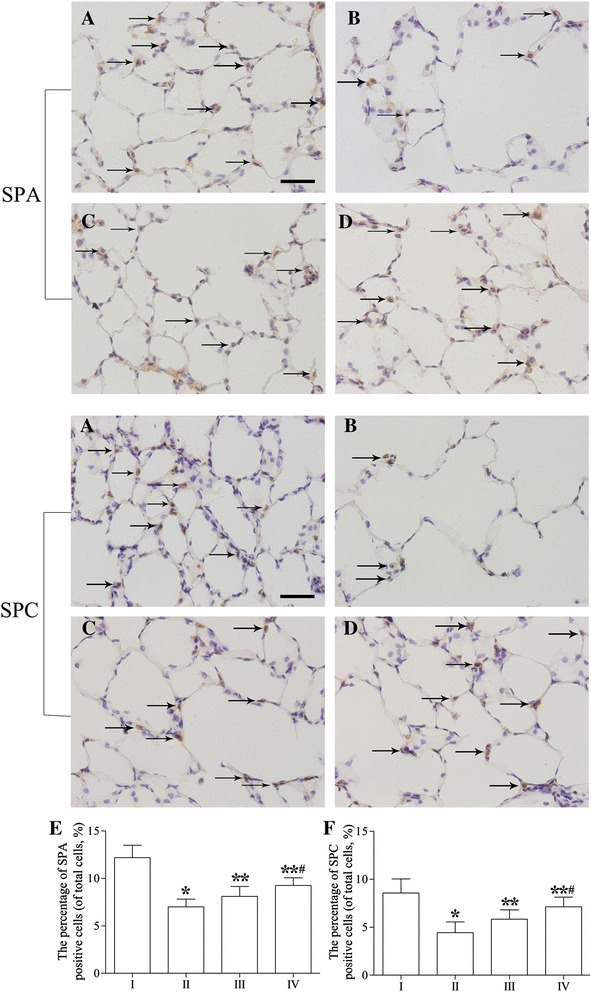
**Expression of SPA and SPC proteins examined by means of immunohistochemical staining. (A)** Control group (group I). SPA and SPC staining was strongly positive. **(B)** Emphysema group (group II). SPA and SPC were expressed at lower levels than in group I. **(C)** Emphysema + rAFMSC transplantation for 20 days (group III). SPA and SPC expression was increased after rAFMSC transplantation for 20 days. **(D)** Emphysema + rAFMSC transplantation for 40 days (group IV). SPA and SPC expression was increased substantially after rAFMSC transplantation for 40 days. **(E)** Percentages of SPA-positive cells in the 4 groups. The percentage of SPA-positive cells was significantly lower in group II than in group I (*P* < 0.05), but the percentage was markedly increased after rAFMSC transplantation for 20 and 40 days (*P* < 0.05). **(F)** Percentages of SPC-positive cells in the 4 groups. The percentage of SPC-positive cells was significantly lower in group II than in group I (*P* < 0.05), but the percentage was markedly increased after rAFMSC transplantation for 20 and 40 days (*P* < 0.05). Values are presented as average percentages of SPA- and SPC-positive cells (means ± SEM, n = 10); * *P* < 0.05 versus group I, ** *P* < 0.05 versus group II, ^#^ *P* < 0.05 versus group III. Scale bars, 50 μm.

After immunohistochemical staining, SPA- and SPC-positive cells exhibited a yellowish-brown color in the cytoplasm, and these cells were mainly located in the alveolar corner of AECII (Figure 6A-D). The expression of SPA and SPC was weaker in group II (Figure 6B) than in group I (Figure 6A). However, after rAFMSC transplantation (groups III and IV), the expression of SPA and SPC increased significantly compared with that in the emphysema group (group II), especially in the case of group IV. Similarly, the percentages of SPA- and SPC-positive cells were markedly lower in group II (7.0% ± 0.8% and 4.4% ± 1.1%) than in group I (12.2% ± 1.2% and 8.5% ± 1.4%) (95% confidence intervals, SPA, −6.11% ~ −4.28%; SPC, −5.17% ~ −3.07%; *P* < 0.05; Figure 6E,F). However, the percentages of SPA- and SPC-positive cells were significantly higher in group III (8.1% ± 1.0% and 5.8% ± 0.9%) and group IV (9.2% ± 0.8% and 7.1% ± 1.0%) than in group II (95% confidence intervals, group III, SPA, 0.18% ~ 2.01%; SPC, 0.35% ~ 2.44%; group IV, SPA, 1.33% ~ 3.16%; SPC, 1.62% ~ 3.72%; *P* < 0.05), especially in group IV (95% confidence intervals, SPA, 0.23% ~ 2.06%; SPC, 0.22% ~ 2.32%; *P* < 0.05). These results indicate that after transplantation, rAFMSCs might have differentiated into AECII-like cells in the lung tissues of rats of groups III and IV, and expressed SPA and SPC.

### Quantification of SPA and SPC expression

**Figure 7:**
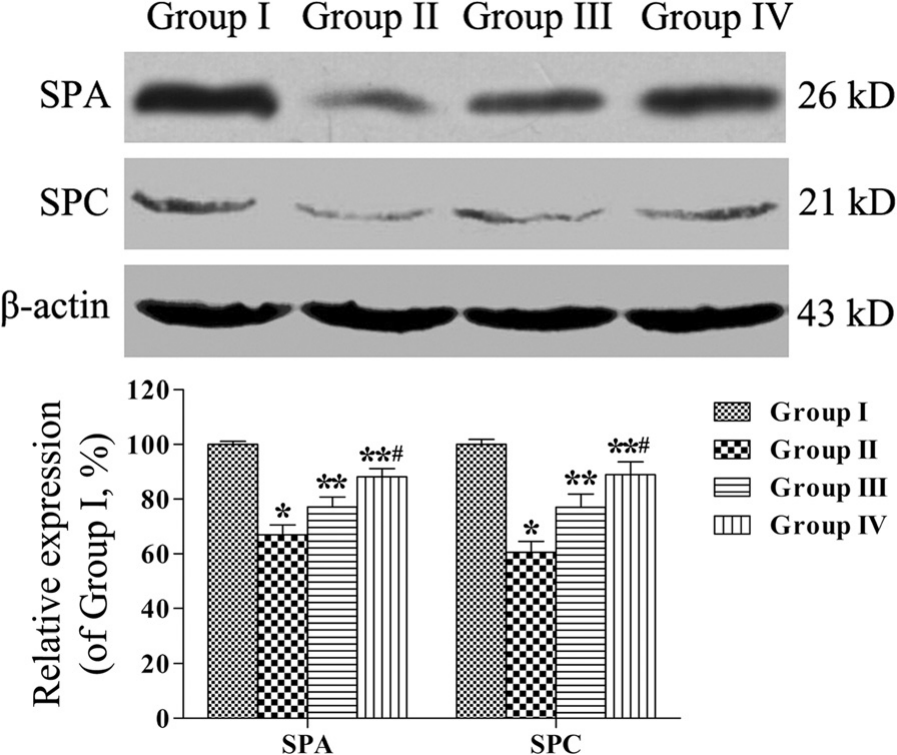
**Quantification of SPA and SPC protein expression by means of western blotting analysis.** The loading control used was β-actin. The expression of SPA and SPC proteins was analyzed by performing densitometry and normalizing the values relative to that of β-actin. All analyses were performed in triplicate. Values are presented as means ± SEM (n = 10). *P < 0.05 versus group I, **P < 0.05 versus group II, ^#^ *P* < 0.05 versus group III.

SPA and SPC expression was quantified by performing western blotting analysis. The results in Figure 7 show that the expression of SPA and SPC was lower in the emphysema group (66.96% ± 3.54% and 60.58% ± 3.96%) than in the control group (95% confidence intervals, SPA, −36.98% ~ −29.10%; SPC, −46.84% ~ −32.00%; *P* < 0.05). Conversely, rAFMSC treatment increased the expression levels of SPA and SPC (group III, 77.16% ± 3.59% and 76.03% ± 4.86%; group IV, 88.15% ± 3.00% and 87.90% ± 4.74%) as compared with the expression in group II (95% confidence intervals, group III, SPA, 6.26% ~ 14.14%; SPC, 9.03% ~ 23.87%; group IV, SPA, 17.25% ~ 25.13%; SPC, 20.89% ~ 35.74%; *P* < 0.05), especially in group IV (95% confidence intervals, SPA, 7.05% ~ 14.93%; SPC, 4.44% ~ 19.29%; *P* < 0.05).

### *Sry* mRNA expression

**Figure 8:**
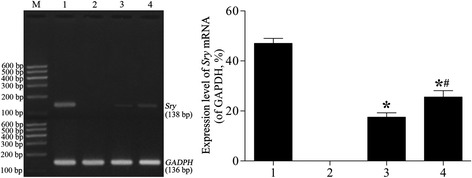
***Sry*** **mRNA expression.** M, DNA ladder marker; Lane 1, positive-control group (male rats); Lane 2, control group (female rats); Lane 3, emphysema + rAFMSC transplantation for 20 days (group III); Lane 4, emphysema + rAFMSC transplantation for 40 days (group IV). The expression of *Sry* mRNA was analyzed by normalizing the values relative to that of GAPDH. Values are presented as average expression levels of *Sry* mRNA (means ± SEM, n = 10); *P < 0.01 versus positive-control group, ^#^ *P* < 0.01 versus group III.

To determine whether rAFMSCs integrated into lung tissues after transplantation, we used RT-PCR to analyze the expression of *Sry* mRNA (Figure 8). When rAFMSCs were transplanted for 20 days (group III), the expression of *Sry* mRNA could be detected, and the expression level was increased significantly in group IV (*t* = −5.768; *P* < 0.01). However, *Sry* mRNA was not expressed in groups I and II, but it was strongly expressed in a positive-control group (male rats).

### Apoptosis of type II alveolar epithelial cells

**Figure 9:**
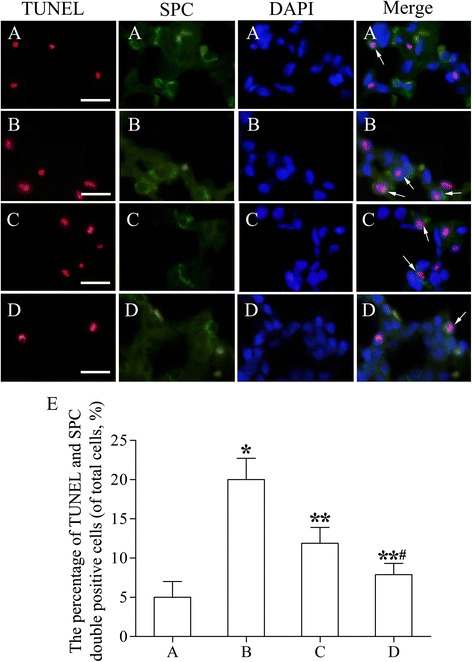
**Apoptosis of AECII in lung tissues. (A)** Control group (group I). TUNEL (red) and SPC (green) double-positive cells (apoptotic AECII) were detected infrequently. **(B)** Emphysema group (group II). Numerous apoptotic AECII were detected. **(C)** Emphysema + rAFMSC transplantation for 20 days (group III). The number of apoptotic AECII was decreased after rAFMSC transplantation for 20 days. **(D)** Emphysema + rAFMSC transplantation for 40 days (group IV). The number of apoptotic AECII was decreased substantially. Cells were counterstained with DAPI. **(E)** The number of apoptotic AECII in each group. Values are presented as the average apoptotic-AECII numbers (means ± SEM, n = 10); *P < 0.01 versus group I, **P < 0.01 versus group II, ^#^ *P* < 0.01 versus group III. Scale bars, 40 μm.

AECII apoptosis was measured using TUNEL and SPC double-immunofluorescence staining. Apoptotic AECII exhibited red fluorescence in the cell nuclei (TUNEL-positive) and green fluorescence in the cytoplasm (SPC-positive) (Figure 9A-D). The percentage of AECII apoptosis in group II was significantly higher than that in group I (95% confidence intervals, 12.96% ~ 16.44%; *P* < 0.01; Figure 9E). After rAFMSC transplantation (groups III and IV), the apoptosis percentages were markedly lower than that in group II (95% confidence intervals, group III, −9.94% ~ −6.46%; group IV, −13.74% ~ −10.26; *P* < 0.01), especially in group IV (95% confidence intervals, −5.54% ~ −2.06%; *P* < 0.01). These results indicated that AECII apoptosis decreased in rats after rAFMSC transplantation.

### Differentiation of rAFMSCs in lung tissues

**Figure 10:**
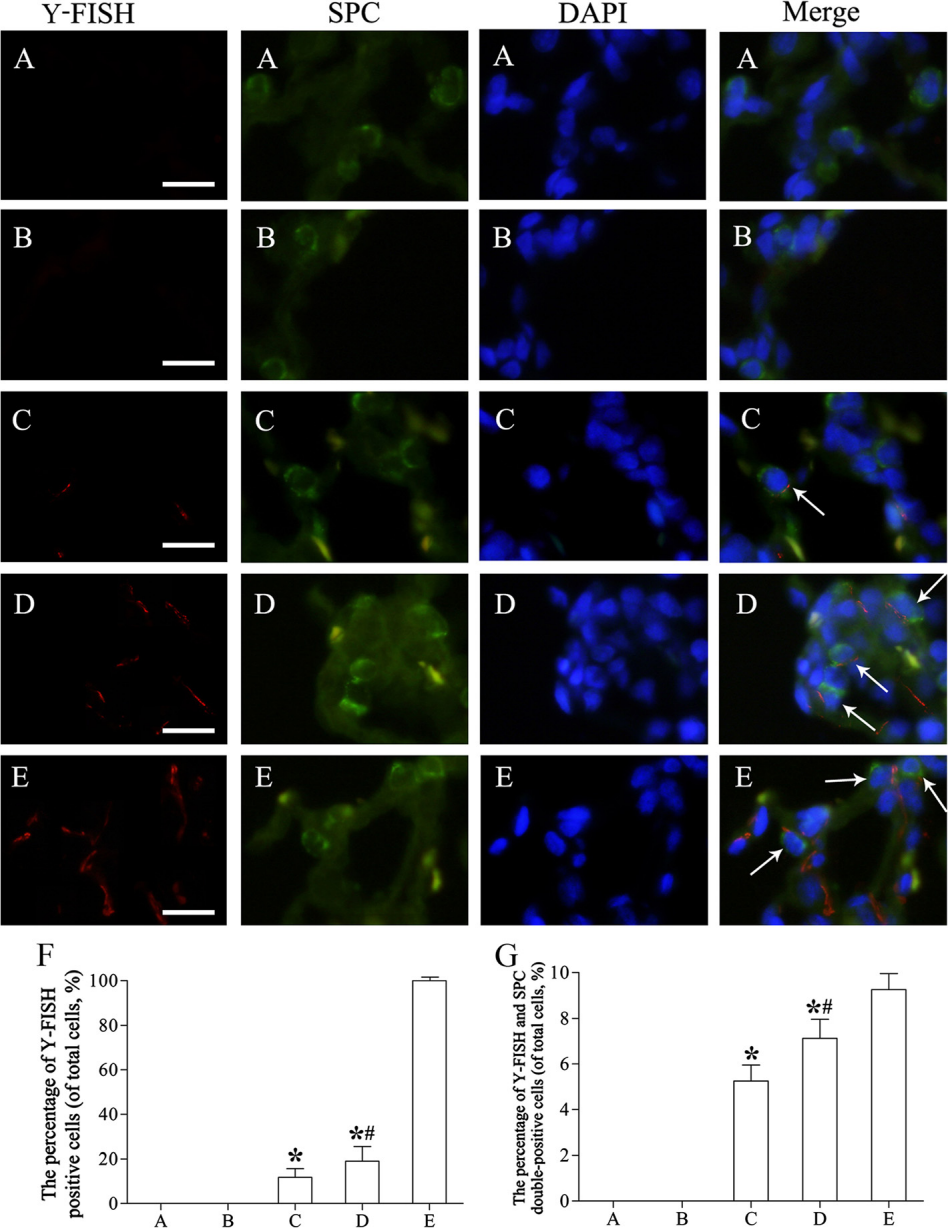
**Y-FISH and SPC double-immunofluorescence staining in lung tissues. (A)** Control group (group I). Y-FISH staining (red) was negative. Nuclei were counterstained with DAPI (blue). **(B)** Emphysema group (group II). Y-FISH staining was again negative. **(C)** Emphysema + rAFMSC transplantation for 20 days (group III). Y-FISH staining was weakly positive and a few Y-FISH-positive cells were also positive for SPC staining (green). **(D)** Emphysema + rAFMSC transplantation for 40 days (group IV). The number of Y-FISH-positive cells was increased substantially, and the number of Y-FISH and SPC double-positive cells was markedly higher than that in group III. **(E)** Positive-control group (male rats). Y-FISH staining was strongly positive. Cells were counterstained with DAPI. **(F)** The percentage of Y-FISH-positive cells in each group. **(G)** The percentage of Y-FISH and SPC double-positive cells in each group. Values are presented as average percentages of positive cells (means ± SEM, n = 10); *P < 0.01 versus group I, ^#^ *P* < 0.05 versus group III. Scale bars, 40 μm.

To further determine how the lung injury caused by emphysema can be repaired by transplanted rAFMSCs after integration, we analyzed Y-FISH and SPC double-immunofluorescence staining in lung sections. The integration of rAFMSCs (Y-chromosome-positive cells) into lung tissues of rats was detected by means of Y-FISH analysis (Figure 10). The results revealed that Y-FISH-positive cells were abundant in the lung sections of male rats (positive-control group; Figure 10E), but the cells were not detected in the sections from the rats of groups I and II (Figure 10A,B). After rAFMSCs were transplanted for 20 days (group III) or 40 days (group IV), the expression of Y-FISH-positive cells was significantly higher than that observed in groups I and II (group III, *t* = −8.327; group IV, *t* = −8.209; *P* < 0.01; Figure 10C,D). Furthermore, the percentage of Y-FISH-positive cells in group IV was significantly higher than that in group III (*t* = −2.675; *P* < 0.05; Figure 10F), which agrees with the *Sry* mRNA-expression results. These results revealed that the rAFMSCs had integrated into the lung tissues of the female rats in groups III and IV after being transplanted for 20 and 40 days, respectively.

To determine whether the rAFMSCs differentiated into AECII-like cells after transplantation, we examined the expression of SPC in Y-FISH-positive cells by performing double-immunofluorescence staining (Figure 10). After rAFMSCs were transplanted into lung tissues for 20 days (group III), Y-FISH and SPC double-positive cells were detected under the fluorescence microscope, and the percentage of these double-positive cells in the sections of the rats of group IV was higher than that measured in the case of group III (*t* = −3.266; *P* < 0.05; Figure 10G).

## Discussion

No drugs are currently available for reversing the progression of COPD and the chronic airflow limitation and emphysema associated with it. Therefore, novel techniques that can be used for effectively treating COPD must be explored. Cigarette smoking is the primary risk factor for COPD, which is related to the inflammatory reactions of lung tissues [44]. LPS, which is a structural component of the outer membrane of gram-negative bacteria, plays key roles in injuring the epithelium and activating inflammatory cells to secrete inflammatory factors and proteases, which contribute to the pathogenesis of chronic bronchitis and emphysema [45]. In this study, emphysema was induced in rats by exposure to cigarette smoke and LPS.

Over the past few decades, stem cells have been used in regenerative medicine [27,33,36,46]. Previously, AFSCs were shown to differentiate into lung epithelial cells after implantation into lung tissue *in vivo* [33]. AFMSCs, which can be readily isolated from amniotic fluid, are multipotent stem cells that exhibit the characteristics of MSCs [35,36]. In this study, we isolated and identified rAFMSCs, which were derived from fetal male rats, and we showed that after transplantation, rAFMSCs could alleviate the lung injury caused by emphysema.

We isolated and purified rAFMSCs and analyzed their phenotypic characteristics, and the results revealed that the rAFMSCs did not express the surface antigens CD14, CD19, CD34, and CD45, but strongly expressed CD29, CD44, CD73, CD90, CD105, and CD166; this agrees with the results of Pan et al. [36]. Oct-4, a POU transcription factor, is a marker of pluripotent stem cells [47] that disappears quickly after cells differentiate [48]. Oct-4 has been reported to be expressed in embryonal carcinoma cells, ESCs, embryonic germ cells, and amniotic fluid stem cells, and to play a vital role in determining the fate of stem cells [49-51]. Here, *Oct-4* mRNA was expressed in rAFMSCs at considerably higher levels than in rat lung fibroblasts, which revealed that rAFMSCs exhibit the stem cells’ characteristics for differentiation. The isolated rAFMSCs could be induced to differentiate into adipocytes and osteocytes, which demonstrated that the differentiation potential of rAFMSCs was similar to that of AFSCs [28,29].

We transplanted rAFMSCs into rats for 20 and 40 days by means of intratracheal instillation and then examined the morphological changes in lung tissue. The results revealed that the MLI and MAA measured in lung tissues were markedly improved in groups III and IV when compared with group II (emphysema group). Thus, we hypothesize that rAFMSC transplantation can be used for repairing the lung injury caused by emphysema, and that this transplantation stimulates the expression of pulmonary SPs in rats with emphysema.

SPs include SPA, SPB, SPC, and SPD and are synthesized and secreted by AECII in lung tissue. SPA and SPC, which are secreted specifically by AECII [52,53], play critical roles in reducing the surface tension of alveoli. In this study, lung tissues expressed both SPA and SPC mRNAs and proteins after rAFMSC transplantation (groups III and IV) at levels markedly higher than those in the emphysema group (group II), which agreed with the morphological changes observed. TTF1 is vital for the induction of respiratory cells, including AECII, and it is expressed in lung tissues after AFSC transplantation [33,54]. Here, the *TTF1* mRNA level in groups III and IV was considerably higher than that in group II.

Sry is the Y-chromosome-specific gene that determines the sex of animals [33]. Our results showed that *Sry* mRNA was strongly expressed after rAFMSC transplantation (groups III and IV), but it was not expressed in the absence of transplantation (groups I and II), which indicated that the transplanted rAFMSCs had integrated into lung tissues. Moreover, when we examined AECII apoptosis, the results showed that the percentage of TUNEL and SPC double-positive cells (apoptotic AECII) after rAFMSC integration was substantially lower than the percentage in the emphysema group.

To locate and count the AECII-like cells that were differentiated from the rAFMSCs in lung tissues, we performed immunostaining and observed SPC expression by rAFMSCs, and the identity of these cells was confirmed by means of Y-FISH in the lung tissues. The results indicated that rAFMSCs in the lung tissues might be able to differentiate into AECII-like cells or induce local regeneration of the lung alveolar epithelium after transplantation.

## Conclusions

The results of this study suggest that transplanted rAFMSCs can alleviate the lung injury caused by emphysema; they do so by integrating into lung tissues, potentially differentiating into AECII-like cells or inducing local regeneration of the lung alveolar epithelium, inhibiting AECII apoptosis, and elevating the levels of *SPA*, *SPC*, and *TTF1* mRNAs and SPA and SPC proteins. Thus, rAFMSC transplantation can potentially be used as a method for the treatment of COPD and in lung regenerative therapy.

## Abbreviations

COPD: Chronic obstructive pulmonary disease
AECI: Type I alveolar epithelial cells
AECII: Type II alveolar epithelial cells
AFMSCs: Amniotic fluid-derived mesenchymal stromal cells
ESCs: Embryonic stem cells
rAFMSCs: Rat amniotic fluid-derived mesenchymal stromal cells
Oct-4: Octamer-binding transcription factor 4
SPs: Surfactant proteins
SPA: Surfactant protein A
SPB: Surfactant protein B
SPC: Surfactant protein C
SPD: Surfactant protein D
TTF1: Thyroid transcription factor 1
BMMSCs: Bone marrow-derived mesenchymal stromal cells
AFSCs: Amniotic fluid-derived stromal cells
MSCs: Mesenchymal stromal cells
DMEM: Dulbecco's modified Eagle's medium
bFGF: Basic fibroblast growth factor
FITC: Fluorescein isothiocyanate
PE: Phycoerythrin
MLI: Mean linear intercept
MAA: Mean alveolar airspace
GAPDH: Glyceraldehyde-3-phosphate dehydrogenase
RT-PCR: Reverse transcription PCR
TUNEL: Terminal deoxynucleotidyl transferase dUTP-mediated nick end labeling
FISH: Fluorescence *in situ* hybridization
Y-FISH: Fluorescence *in situ* hybridization for Y chromosome
